# Plant and pathogen genomics: essential approaches for stem rust resistance gene stacks in wheat

**DOI:** 10.3389/fpls.2023.1223504

**Published:** 2023-09-01

**Authors:** Matthias Jost, Megan A. Outram, Kathy Dibley, Jianping Zhang, Ming Luo, Michael Ayliffe

**Affiliations:** Commonwealth Scientific and Industrial Research Organisation (CSIRO) Agriculture and Food, Canberra, ACT, Australia

**Keywords:** avirulence, durable, polygenic, plant, disease, resistance, gene

## Abstract

The deployment of disease resistance genes is currently the most economical and environmentally sustainable method of crop protection. However, disease resistance genes can rapidly break down because of constant pathogen evolution, particularly when they are deployed singularly. Polygenic resistance is, therefore, considered the most durable, but combining and maintaining these genes by breeding is a laborious process as effective genes are usually unlinked. The deployment of polygenic resistance with single-locus inheritance is a promising innovation that overcomes these difficulties while enhancing resistance durability. Because of major advances in genomic technologies, increasing numbers of plant resistance genes have been cloned, enabling the development of resistance transgene stacks (RTGSs) that encode multiple genes all located at a single genetic locus. Gene stacks encoding five stem rust resistance genes have now been developed in transgenic wheat and offer both breeding simplicity and potential resistance durability. The development of similar genomic resources in phytopathogens has advanced effector gene isolation and, in some instances, enabled functional validation of individual resistance genes in RTGS. Here, the wheat stem rust pathosystem is used as an illustrative example of how host and pathogen genomic advances have been instrumental in the development of RTGS, which is a strategy applicable to many other agricultural crop species.

## Introduction

Plant disease resistance is not a single mechanistic process but consists of multiple defense mechanisms, both pre-anticipatory and inducible (Jones and Dangl, 2006). Contributing to these defense mechanisms are two broad categories of resistance genes: qualitative resistance genes (*R*) and quantitative resistance genes (*QR*). *R* genes often encode immune receptor proteins that contain a central nucleotide binding domain, a C-terminal leucine-rich repeat domain, and a variable N-terminal domain (NLR proteins) (Chen et al., 2022; Maruta et al., 2022). These almost-ubiquitous plant receptors detect pathogen attack by each recognizing one of many pathogen virulence molecules (effectors) that are introduced into a plant cell to facilitate parasitism. Upon pathogen effector recognition, a plant defense response is activated, which often culminates in death of the infected host cell. Molecular recognition can be either a direct association between the NLR protein and corresponding recognized effector [called an avirulence effector (Avr)], an NLR/Avr association in a host–protein complex, or by NLR recognition of Avr-mediated modification of a host–protein (guardee hypothesis) (Jones and Dangl, 2006; Chen et al., 2022; Maruta et al., 2022). *R* gene products are not limited to NLR proteins and include tandem protein kinases and wall-associated kinases. The underlying molecular mechanisms leading to resistance for these latter molecules are less clear (Sanchez-Martin and Keller, 2021), but their race specificity (in some instances) implies that they are part of an effector-based recognition process, possibly by acting as guardee effector targets (Jones and Dangl, 2006). In the case of wheat stem rust disease caused by the fungal pathogen *Puccinia graminis* f.sp. *tritici* (*Pgt*), 15 wheat *R* genes have been cloned, 12 of which encode NLR proteins and three that encode protein kinases (**Table 1**).

**Table 1:** Cloned wheat stem rust resistance genes.

Gene	Origin	Product	Resistance	Reference
*Sr13*	*Triticum turgidum* ssp. *durum*	NLR	R	Zhang et al., 2017
*Sr21*	*T. monococcum*	NLR	R	Chen et al., 2018
*Sr22*	*T. monococcum* ssp. *boeoticum*	NLR	R	Steuernagel et al., 2016
*Sr26*	*Thinopyrum ponticum*	NLR	R	Zhang et al., 2021
*Sr27*	*Secale cereale*	NLR	R	Upadhyaya et al., 2021
*Sr33*	*Ae. tauschii*	NLR	R	Periyannan et al., 2013
*Sr35*	*T. monococcum*	NLR	R	Saintenac et al., 2013
*Sr43*	*Thinopyrum elongatum*	Protein kinase	R	Yu et al., 2023
*Sr45*	*Ae. tauschii*	NLR	R	Steuernagel et al., 2016
*Sr46*	*Ae. tauschii*	NLR	R	Arora et al., 2019
*Sr50*	*Secale cereale*	NLR	R	Mago et al., 2015
*Sr55/Lr67/Yr46/Pm46/Ltn3*	*T. aestivum*	Hexose transporter	QR	Moore et al., 2015
*Sr57/Lr34/Yr18/Pm38/Ltn1*	*T. aestivum*	ABC transporter	QR	Krattinger et al., 2009
*Sr60*	*T. monococcum*	Tandem kinase	R	Chen et al., 2020
*Sr61*	*Thinopyrum ponticum*	NLR	R	Zhang et al., 2021
*Sr62*	*Ae. sharonensis*	Tandem kinase	R	Yu et al., 2022
*SrTA1662*	*Ae. tauschii*	NLR		Gaurav et al., 2022

Individual *R* proteins can often provide very high levels of disease resistance, and their monogenic inheritance makes them ideal for breeding use. However, when deployed singularly, these *R* genes are often rapidly overcome because of pathogen evolution. Specifically, the pathogen can mutate or lose individual recognized avirulence effector molecules to avoid *R* protein–mediated recognition and subsequent plant defense activation. In practice, pathogen populations often consist of isolates that carry an avirulence effector and isolates carrying a modified (or deleted) unrecognized version of the effector (virulence effector), which enables them to avoid *R* protein detection, leading to successful pathogen virulence. Given the regional genetic variation of effector genes in different pathogen populations, an *R* protein may be valuable in one region but of limited use in another. Polygenic deployment of *R* genes is, therefore, the most effective as multiple pathogen effectors are simultaneously recognized, which also enhances individual *R* gene durability as multiple pathogen effector gene mutations are then needed for pathogen virulence. However, multigenic resistance breeding is complex as *R* genes are generally unlinked making creating and maintaining *R* gene combinations in breeding programs a difficult and laborious task.

In contrast, quantitative resistance (*QR*) genes provide moderate to minor levels of partial disease resistance that is often singularly insufficient to provide agronomically acceptable disease protection (Poland et al., 2009; Niks et al., 2015; Kumar and Nadarajah 2020; Delplace et al., 2020). However, these minor loci can be additive and, when combined in sufficient numbers, can provide high levels of resistance (Singh et al., 2000; Singh et al., 2011; Singh et al., 2015; Fukuoka et al., 2015; Huerta-Espino et al., 2020). Note that, in some cases, quantitative resistance occurring later in plant development can also be conferred by *NLR* genes. One such example is the wheat leaf rust *Lr22a* resistance gene, which encodes an NLR family protein that likely functions by effector recognition (Thind et al., 2017). These types of *NLRs* are not discussed as *QR* genes in this review.

Mechanistically, *QR* is less well understood; however, effective polygenic quantitative resistance is considered more durable than *R* gene–mediated resistance (Parlevliet, 2002; Niks et al., 2015; Pilet-Nayel et al., 2017; Cowger and Brown, 2019; Dinglasan et al., 2022). In some instances, a single quantitative *R* gene can have remarkable durability, a case in point being the wheat *Sr57/Lr34/Yr18/Pm38/Ltn1* gene (hereafter, *Sr57*) that has been used for many decades in agriculture and not overcome (Dyck et al., 1966; Kolmer et al., 2008). This gene provides partial resistance only in adult wheat plants, but it is broadly effective against stem rust, leaf rust, stripe (yellow) rust, and powdery mildew diseases (Krattinger et al., 2009; Moore et al., 2015). The *Sr57* gene encodes a variant form of a membrane-bound ABC transporter that is suggested to be involved in abscisic acid transport (**Table 1**).

Other cloned wheat *QR* genes include the *Sr55/Lr67/Yr46/Pm46/Ltn3* gene (hereafter, *Sr55*) (**Table 1**) that, like *Sr57*, is effective against all three wheat rust pathogens and powdery mildew disease and encodes a hexose transporter (Krattinger et al., 2009; Moore et al., 2015). The distinctly different structure of both these membrane transporter *QR* proteins compared with *R* proteins suggests very different modes of actions, which may explain the contrasting durability and efficacy of the two resistance types. In contrast, the wheat *Yr36* gene confers *QR* to wheat stripe rust only (Fu et al., 2009) and encodes a steroidogenic acute regulatory protein-related lipid transfer (START) domain containing kinase protein that gives it some structural similarity to tandem kinase *R* proteins such as *Sr60* and *Sr62* (**Table 1**). The Yr36 protein is believed to interact with a chloroplast peroxidase protein, resulting in elevated levels of hydrogen peroxide production (Fu et al., 2009; Gou et al., 2015).

Polygenic deployment of both *R* and *QR* genes is, therefore, highly desirable for improving resistance efficacy and durability. An effective approach to circumvent the genetic complexity of polygenic resistance is to combine cloned *R* or *QR* genes onto a single–*Agrobacterium* transferred-DNA (T-DNA) molecule and then to introduce these genes into the plant genome as a single insertion. These resistance “transgene stacks” (RTGS) reduce the genetic complexity of multiple transgenes to monogenic inheritance, which greatly simplifies subsequent gene deployment in breeding programs. RTGS combinations of *R* and *QR* genes are also considered highly desirable in terms of efficacy and durability.

Here, RTGS are defined as a single locus produced by plant transformation that encodes three or more transgenes that are either *R* genes, *QR* genes, or *R* + *QR* gene combinations. Previously, the terms gene stacking and pyramiding have been used to describe the combining of multiple unlinked resistance loci by conventional plant breeding (Joshi and Nayak, 2010). Alternatively, combining linked resistance genes in *cis* on a single chromosome segment by conventional recombination has also been referred to as creating gene stacks (Zhang et al., 2021). The term RTGS differentiates this approach to these other conventional breeding approaches by highlighting the transgenic (T) nature and, hence, single-locus inheritance of the event.

Other advantages of RTGS are the prevention of single–*R* gene deployment and consequent erosion of these valuable genetic resources. Once produced, a RTGS requires no more breeding effort than a single *R* gene yet offers broad-spectrum resistance that is potentially more durable. Using cloned genes in RTGS also negates linkage drag due to the precise and minimal nature of each gene sequence, in contrast to resistance genes residing on chromatin segments, particularly those from wild relatives that can show poor recombination with the wheat genome (Wulff and Moscou, 2014). Herein, the development of RTGS in crop plants is described, with an emphasis on the authors’ experience in developing wheat RTGSs that provide resistance to wheat stem rust. This example is used to highlight the underlying advances in genomic technologies that have expanded the availability of cloned plant resistance gene for RTGS production and also increased the isolation of corresponding pathogen avirulence effectors that, when available, can be used to confirm individual *R* gene function in RTGS.

## Genomic technologies for cloning *R* genes

**Figure 1:**
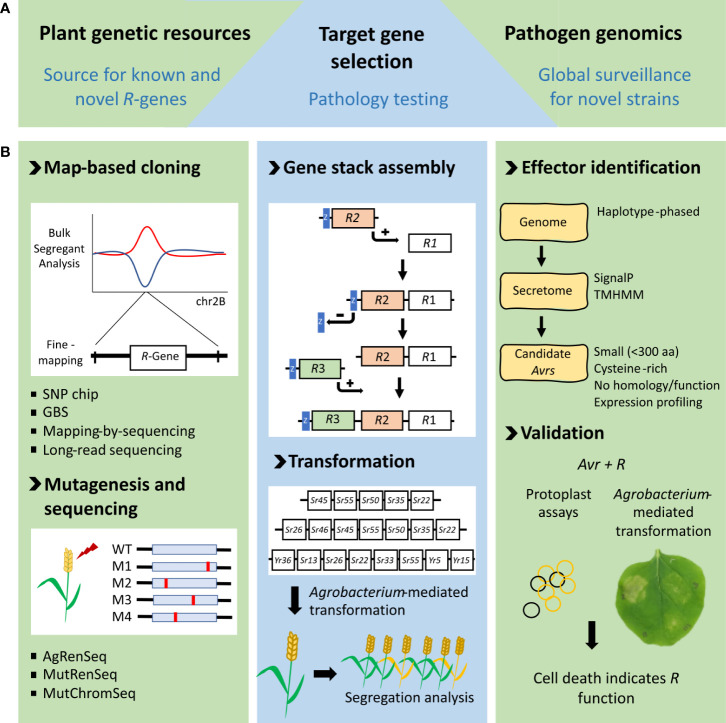
Pipeline of resistance gene stacks (RTGS) development. **(A)** Resources required for successful RTGS design include *R* genes from plant genetic resources, surveillance of rust strains and detailed pathology testing to produce the most broadly effective RTGS. **(B)** Methodology for generating RTGS. The left panel shows genetic and mutagenesis-based approaches for *R*-gene discovery. The middle panel shows the gene stack assembly process where an R-gene in a donor vector with a linked *lacZ* reporter (blue box) is introduced into a binary destination vector by Gateway recombination. *lacZ* is removed to re-create the destination vector before addition of the next *R*-gene (Luo et al, 2019). Examples of rust *R* gene stacks we have created and transformed into wheat are shown beneath. Segregation analysis of T1 progeny is used to confirm all genes are co-inherited. The panel on the right shows the current workflow for identifying new rust *Avrs*. This process is underpinned by highly accurate genome assemblies, made possible with new long-read sequencing technologies. Effector proteins are generally secreted by the pathogen into the host where they function, making small, secreted pathogen proteins prime avirulence effector candidates. Avirulence effectors are confirmed by cell death assays using either protoplasts or transient leaf expression assays with the corresponding *R* protein.

Resistance gene cloning from wheat has been hampered by the large size of its highly complex, repetitive, and polyploid genome. However, some major advances in genomic technologies described below have facilitated the isolation of both *R* and *QR* genes from wheat and numerous other plant species (**Figure 1**).

## Target enrichment sequencing

Targeted gene enrichment sequencing approaches have considerably shortened the *R* gene isolation process from plant genomes. *R* gene enrichment Sequencing (RenSeq) is an *R* gene target enrichment platform that, in the case of wheat, uses a bait library of known *NLR* sequences from the *Triticeae* family for sequence capture (Jupe et al., 2013). Captured sequences from a cultivar of interest are then deep-sequenced to efficiently characterize the majority of *NLR* sequences present in its genome. When combined with chemical mutagenesis, in a process called MutRenSeq, causative *R* genes can be identified by comparing *R* gene complements of parent and mutant derivatives (Steuernagel et al., 2016). RenSeq-captured *NLR* sequences are interrogated for an *R* gene mutated in all susceptible derivatives to identify the causative gene. This approach has enabled isolation of wheat stem rust *R* genes *Sr22*, *Sr26*, *Sr45*, and *Sr61* (**Table 1**). A further modification of this approach, AgRenSeq, negates the need for mutants by combining association genetics and RenSeq to screen germplasm collections for causative *NLR* resistance genes (Arora et al., 2019; Kale et al., 2022).

However, one limitation of exome-based sequence capture methods is their inability to isolate regulatory sequences that are required for RTGS and can be technically challenging to identify. In addition, RenSeq approaches make an *a priori* assumption that resistance is encoded by an *NLR* gene that, as described above, is not always the case. In contrast, the more laborious process of conventional positional cloning does not rely upon this assumption. A significant advance over positional cloning has been MutChromSeq. This approach, like RenSeq, also uses sequence complexity reduction; however, it does not make underlying causative gene identity assumptions. In MutChromSeq, chromosomes of wild-type and mutant progeny are flow-sorted and the chromosomes encoding the resistance gene of interest are deep-sequenced from each plant (Sanchez-Martin et al., 2016). The causative resistance gene is again identified by being mutated in all susceptible plants relative to the wild-type, parental sequence. MutChromSeq is potentially applicable for both *R* and *QR* gene isolation; however, it requires the chromosomal location of the resistance gene to be known.

## Mining wheat germplasm for novel *R*-genes

Unimproved plant genetic resources, like wild relatives and landraces, can be valuable sources of new resistance genes, and these genes have been isolated using the positional cloning and targeted sequence enrichment approaches described above. Near entire gene complements from large germplasm collections have also been identified using untargeted complexity reduction methods coupled with large-scale sequencing. Restriction site–based genotyping-by-sequencing (GBS) approaches have been used to characterize two large germplasm collections of wheat: 80,000 wheat accessions (Sansaloni et al., 2020) and 8,000 winter wheat varieties, respectively (Schulthess et al., 2022), and these analyses have identified untapped genetic diversity for potential future crop improvement. GBS also helps to reduce redundancy in germplasm collections, enabling the establishment of smaller core collections that capture maximum genetic diversity.

Representative genetic panels have been screened for rust response, enabling resistance locus identification by GWAS, including new resistances not previously used in wheat breeding. For example, screening the 8,000 accessions present in the winter wheat collection with stripe rust identified a trait-specific collection of 150 resistant genotypes and 50 susceptible genotypes (Schulthess et al., 2022). This core stripe rust resistance collection was then whole-genome shotgun–sequenced with three times coverage to identify 23 putatively novel stripe rust resistance genes (Schulthess et al., 2022). In a similar approach, the stem rust gene *SrTA1662* was cloned by *kmer* based association genetics of a whole-genome–sequenced diversity panel of 242 *Ae. tauschii* accessions (Gaurav et al., 2022).

## The role of the wheat pan-genome

Integral to these sophisticated targeted and untargeted complexity reduction approaches and advanced positional cloning methods has been the development of a high-quality wheat pan-genome. This genome sequence greatly assists comparative genetic studies for resistance gene identification including Genome-wide association studies (GWAS), quantitative trait loci (QTL) mapping, and bi-parental genetic mapping approaches. Since the first complete high-quality reference genome of spring wheat cultivar Chinese Spring (International Wheat Genome Sequencing, 2018), a further 10 chromosome-scale reference assemblies and five long scaffold assemblies have been produced, providing a valuable insight into wheat structural genomic variation (Walkowiak et al., 2020). These sequenced wheat genomes also have significant variation among *NLR* gene families with copy number and presence/absence variations and some *NLRs* present only in wild ancestors. Among 10 wheat genomes, a common set of *NLRs* was identified that comprised only 31%–34% of total *NLR* genes present, which highlights the *R* gene diversity that exists between accessions (Walkowiak et al., 2020). Therefore, the current wheat pan-genome, while being an essential genomics tool, does not yet sufficiently represent the diversity of *R* genes present in wheat and wheat relatives. Even less clear is how representative the current wheat pan-genome is for *QR* genes given the paucity of these cloned genes and their structural diversity. Nonetheless, the pan-genome remains an essential tool for resistance gene isolation and greatly enhances molecular-genetic and sequence-based approaches.

More conventional molecular-genetic approaches like bulk segregant analysis (Michelmore et al., 1991) can be combined with high-throughput sequencing (Bulked Segregant Analysis by sequencing (BSA-seq)) and pan-genome homology to allow the rapid assignment of resistance loci to chromosomal locations. Sequencing of bulked DNA samples from pools of phenotypically resistant and susceptible sibs enables rapid mapping of resistance loci based on single nucleotide polymorphism (SNP) allele frequencies (Takagi et al., 2013; Mascher et al., 2014). As this approach can tolerate some minor phenotypic inconsistencies, it is particularly useful for mapping resistance phenotypes challenging to score, for instance, quantitative resistances. For example, mapping of the incompletely dominant wheat stripe rust resistance gene *Yr84*, of wild emmer wheat, to chromosome 1BS was achieved using only a small segregating F2 population of 92 individuals (Klymiuk et al., 2022). Although the mapping interval size depends upon several factors, including phenotypic accuracy, numbers of genotypes per bulk, sequencing depth, and recombination frequency in the target area, BSA-seq remains a robust tool for rapidly assigning loci to chromosomal locations.

Fine mapping is an essential tool for distinguishing between multiple resistances that are located in similar chromosomal regions, and the wheat pan-genome greatly assists marker development for fine mapping regions of interest. SNP variants from BSA-seq can be readily converted to markers to further refine the genetic interval by high-resolution mapping using larger families (Klymiuk et al., 2022). Combining high marker density with genome sequencing and pan-genome alignment enables the assembly of a contiguous sequence spanning the resistance locus. However, this is still not a trivial process given the large size of the wheat genome. For example, cloning of the *Sr62* tandem kinase gene from the diploid wheat relative *Aegilops sharonensis* (**Table 1**) required a reference sequence using short-read Illumina data to be combined with chromosome sorting, different length mate-paired libraries, 10x genome coverage, and chromosome confirmation capture (Yu et al., 2022).

Technology improvements, however, have enabled *de novo* assembly of wheat genomes and genomic regions that reduce the reliance on the wheat pan-genome. Sequences that span resistance loci have been assembled using long-range scaffolding combined with chromosome capture and sequencing, which can produce assemblies that encode Mbp of complex repetitive regions. For instance, combining targeted chromosome-based long-range assembly (TACCA) and MutChromSeq technology enabled cloning of the wheat *Lr22a* leaf rust resistance gene. This example highlights the value of a high-quality reference sequence that spans the resistance locus, albeit at a chromosome scale in this instance (Thind et al., 2017).

Improvements in sequencing technologies and reductions in sequencing costs are enabling wheat genomes to be sequenced more routinely. Using long-range sequencing, the *Yr27* stripe rust *R* gene was isolated by sequencing and *de novo* assembly of the complete genome of resistant wheat accession Kariega (Athiyannan et al., 2022). PacBio circular consensus sequencing combined with chromosome conformation capture enabled the entire 10-Mbp *Yr27* interval to be sequenced without using genome complexity reduction methods. Loss-of-function mutants were then used for causative gene identification within the interval (Athiyannan et al., 2022). The genome of wheat cultivar Renan, which carries multiple fungal disease resistance-encoding introgressions from *Aegilops ventricosa*, was assembled using the Oxford Nanopore Technology PromethION (Aury et al., 2022). Similarly, a reference genome of the highly transformable wheat cv. Fielder has been produced and is proving to be a valuable experimental resource, particularly for the analysis of gene editing events (Sato et al., 2021). The assembly of high-quality, chromosome-scale genomes can now be achieved in a very short time frame; recently, the genome of wheat cultivar Attraktion was assembled in 3 months at a cost of $40,000 Euros (Kale et al., 2022). With further reductions in sequencing costs and *de novo* assembly options, genomic sequencing of resistant wheat cultivars will become a standard tool for identifying new resistance genes, thereby accelerating the development of new RTGS.

## Resistance transgene stacks

The production of polygenic transgene constructs to enhance plant disease or pest resistance has a long history. In the first examples, combinations of two or, sometimes, more defense-related genes (as opposed to resistance genes) that encoded pathogenesis-related proteins such as glucanase, chitinases, proteinases, or *Bacillus thuringensis* Cry proteins were introduced into plants, although as polyproteins in some cases (Jach et al., 1995; Jongedijk et al., 1995; Urwin et al., 1998; Jha and Chattoo, 2009; Senthilkumar et al., 2010; Quilis et al., 2014; Shehryar et al., 2019). Alternatively, multiple plasmids were co-transformed into plants to combine numerous genes by co-integration (Chen et al., 1998). Polygenic construct approaches became increasingly sophisticated when recombinase cloning systems were introduced to increase construct coding capacity (Dafny-Yelin and Tzfira, 2007). Lin and colleagues (2003) introduced 10 transgenes into rice as a 33-kb construct that included two chitinases: a proteinase inhibitor and the *Xa21* resistance gene. Subsequently, multiple defense-related genes have been simultaneously targeted to the plastid including a sporamin, cystatin, and chitinase gene combination (Chen et al., 2014).

Two NLR genes, *Rpi-blb1* and *Rpi-blb2*, were transformed into potato cultivar Fontane by *Agrobacterium *transformation (Storck et al., 2012), however, the first plant RTGS was developed in potato, when three NLR genes effective against the potato late blight pathogen, *Phytophora infestans*, were combined (Zhu et al., 2012). Susceptible cultivar Desiree was transformed with *Agrobacterium* carrying a 22-kb T-DNA encoding *R* genes *Rpi1-sto1*, *Rpi1-vnt1.1*, and *Rpi-blb3* and a kanamycin selectable marker gene. This RTGS was produced by conventional restriction enzyme–based cloning. *Rpi1-vnt1.1* was shown to function in transgenic potato by infection with a *P. infestans* isolate avirulent to this gene but virulent to the remaining two genes. Function of at least one of the remaining two *R* genes was confirmed by infecting with a second *P. infestans* isolate that was avirulent to both these latter two genes and virulent to *Rpi1-vnt1.1* (Zhu et al., 2012).

These data illustrate how difficult it is to confirm function of each *R* gene in a RTGS, even when only three are present, using differential pathogen isolates. As the number of *R* genes present in a RTGS increases, using differential pathogen isolate specificity to confirm gene function becomes increasingly less feasible. This is, of course, the desired outcome of a RTGS, i.e., to encode multiple R genes that are each effective against as many prevailing pathogen isolates as possible. For the potato RTGS described above, the existence of a *P. infestans* isolate recognized by only a single gene, *Rpi1-vnt1.1* (Zhu et al., 2012), is less than desirable because, in regions where this isolate exists, the RTGS effectively equates to only single–*R* gene deployment. In this potato example, all three *R* genes were subsequently demonstrated as functional in RTGS plants by leaf infiltration with *Agrobacterium* cultures expressing either of the three cognate *P. infestans* effectors, leading to localized cell death (Zhu et al., 2012), an approach discussed in more detail below. Field trialling in Belgium and The Netherlands of Desiree potatoes containing the *Rpi1-sto1*, *Rpi1-vnt1.1*, and *Rpi-blb3* RTGS showed high levels of field resistance to *P. infestans* (Haesaert et al., 2015).

Potato cultivars Desiree and Victoria were subsequently transformed with an 18.5-kb RTGS encoding *P. infestans* resistance genes, *RB (Rpi-blb1)*, *Rpi-blb2*, and *Rpi-vnt1.1*, and a kanamycin resistance selectable marker gene. These lines showed high levels of resistance in field trials in Uganda (Ghislain et al., 2019). This same RTGS was introduced into adapted African potato cultivars Tigoni and Shangi and again high levels of resistance observed using whole-plant and detached leaf assays (Webi et al., 2019).

## Producing wheat RTGS

One of the challenges in producing RTGS is the generally large size of individual plant *R* genes. When coupled with endogenous regulatory sequences (generally 2-kb 5′ and 1-kb 3′ of the translation initiation and termination codons, respectively), each wheat *R* gene is often around 7–8 kb in size. A stack of five wheat genes is, therefore, approximately 40 kb in size (Luo et al., 2021). A Gateway-based reiterative cloning process was used to assemble wheat gene stacks (**Figure 1**; Luo et al., 2021) in preference to Golden Gate cloning, which is usually not practical given the prohibitive amount of sequence domestication required for such large insertions. Key to this cloning strategy is using *lacZ* as a positive selection marker rather than its typical application as a loss-of-function cloning marker by insertional inactivation (**Figure 1**). This latter approach is prone to producing false-positive background colonies arising from imprecise religation of *lacZ*. As gene stacks increase in size the addition of more genes becomes progressively more difficult, making this positive *lacZ* selection strategy even more advantageous. Using this cloning approach, a gene stack 60 kb in size has been generated that encodes eight wheat rust resistance genes (**Figure 1**).

*Agrobacterium*-mediated transformation was used to produced transgenic wheat containing RTGS (Luo et al., 2021) in preference to particle bombardment, to minimize multiple and/or truncated gene insertions. The wheat transformation procedure of Ishida et al. (2014) was used, although cloning vectors and binary vectors with low copy number origins of replication (*P1* for *E. coli* and *pVS1* for *Agrobacterium*) were used throughout. Not unexpectedly, a correlation appears to exist between RTGS size and the recovery of transgenics that have successfully integrated a complete copy of all genes present in the RTGS. From a limited number of experiments, stacks of around 40 kb in size and encoding five genes appear to be optimum for recovering sufficient transgenics that carry all genes, which, in practice, is approximately 5% of primary transgenics (T0) plants produced. Transformation of larger RTGS of 52 kb (seven genes) and 60 kb (eight genes) has been attempted (unpublished), but transgenics containing a complete copy of all genes at a single locus were not recovered (**Figure 1**). Instead, these larger constructs have produced transgenics containing multiple truncated transgene insertions.

Molecular analyses are used to identify transgenics containing a complete RTGS, and subsequent segregation analysis carried out to confirm that all genes are inherited as a single locus. *R* and *QR* gene expression is initially examined by RNA accumulation using either qPCR or, alternatively, for *R* genes, RenSeq (described above) using complementary DNA (cDNA) from transgenic plants (rather than genomic DNA) as a capture target. The latter approach is preferable as this enables the identification of full-length transcripts for each *R* transgene, thereby re-confirming the integrity and expression of each. Given that native *R* gene expression levels are generally low, cDNA sequence enrichment is necessary to ensure adequate transcript coverage of each transgene.

However, transcription does not formally demonstrate gene function, and, as discussed above, using differential pathogen isolates is often not informative for either *R* or *QR* gene function in a RTGS. Previously, the function of the *QR* gene, *Sr55*, was demonstrated in RTGS wheat plants by taking advantage of the multiple pathogen resistance provided by this gene (Luo et al., 2021). The *Sr55* gene (aka *Lr67*) also confers resistance to wheat leaf rust, whereas the remaining genes in the RTGS were effective against stem rust only (i.e., *Sr22*, *Sr35*, *Sr45*, and *Sr50*). In a separate field trial, plants containing this RTGS were shown to have adult plant leaf rust resistance, thereby confirming the function of the *Sr55* gene (Luo et al., 2021). However, this strategy is not applicable for *QR* genes that provide resistance to a single-pathogen species when they are combined with other genes targeting the same species. Confirming *QR* function in this situation is challenging. In the case of additive *QR* genes, the RTGS could be inferred as functional by having increased levels of resistance compared with single-gene controls, although this evidence is only correlative given that different transgene insertions can show different expression levels. Demonstration of single-pathogen *QR* gene function when combined with multiple *R* genes that recognize the same pathogen will require other strategies. In some instances, morphological phenotypes (e.g., leaf tip necrosis) or the induction of specific transcriptional pathways or metabolite profiles may be indicative of a specific *QR* gene function, although knowledge of these secondary transcriptional and metabolic indicators is generally poor.

Currently, the most effective method to confirm individual *R* gene function in RTGS wheat is to individually express cognate effectors in RTGS protoplasts, along with a reporter gene such as *YFP* (Saur et al., 2019; Luo et al., 2021; Arndell et al., 2023). *R* protein effector recognition results in protoplast death and loss of YFP fluorescence. In Solanaceous species like potato, effector expression is more simply achieved using *Agrobacterium* leaf infiltration assays and resistance gene function detected by macroscopic cell death (Zhu et al., 2012). Of course, the ability to carry out either assay requires cognate pathogen effector genes to be available.

Avirulence effectors are becoming more available due to the advances in pathogen genomic technologies described below. However, for both protoplast and leaf infiltration assays, the effector expression levels obtained may not reflect the true biological expression levels encountered during natural infection. Potentially overexpression of an effector could compensate for poor R expression and result in cell death in these artificial assays,which may not be recapitulated during pathogen infection. Nonetheless, protoplast and leaf infiltration assays remain the best available rapid transient *in planta* assays for establishing *R* gene–mediated effector recognition.

## Genomics for pathogen effector isolation

Effectors are key virulence proteins that aid in pathogen infection and colonisation. Typical characteristics of effectors are that they are small (typically <300 amino acids), secreted proteins, rich in cysteine residues. Because of the co-evolutionary arms race between pathogen and host, pathogens constantly modify their effector repertoires to evade host detection while maintaining their virulence functions, as described above. As a result, effectors typically lack conserved motifs or domains of known functions, making their identification a challenging endeavor. For rust pathogens, this is further complicated by their obligate biotrophic lifestyle that prevents conventional experimental tools and strategies, such as transformation and gene knockouts, due to an inability to culture the pathogen *in vitro*. Rust pathogens also have large, highly repetitive genomes compared with most fungal species, i.e., ~80 Mb to ~2 Gb (Tavares et al., 2014; Ramos et al., 2015), posing greater challenges for high-quality genome assembly, which is a prerequisite for effector identification in model and non-model rust systems (Anderson et al., 2016; Chen et al., 2017; Salcedo et al., 2017; Upadhyaya et al., 2021). Like wheat, the number of genome assemblies of rust pathogens has also increased, and reference genomes from approximately a dozen rust species are now available (Figueroa et al., 2020). Largely, this has been possible because of advances in long-read sequencing technologies, such as Pacific Biosciences (PacBio) and Oxford Nanopore Technologies that capture greater amounts of sequence information. Combining these sequencing technologies with new advances in obtaining chromatin contact data, such as Hi-C, has enabled chromosome-level rust genome assemblies to be produced that are complete, gapless, and of high quality. Previous short-read technologies have resulted in highly fragmented assemblies due to the repetitive nature of these genomes that contain from 18% to 75% repeat sequences (Lorrain et al., 2018).

Haplotype-resolved genome assemblies are now available for *Pgt* (Li et al., 2019) and several other economically important rust species (Miller et al., 2018; Schwessinger et al., 2018; Duan et al., 2022; Henningsen et al., 2022; Liang et al., 2023). These assemblies have furthered our understanding of the evolutionary principles driving rust evolution and importantly enabled the large-scale identification of candidate effectors in *silico*. Experimentally validated *Avrs* from rusts and other pathogen species, as discussed above, are generally small-secreted proteins rich in cysteine residues, which enable the bioinformatic identification of genomic complements of candidate effectors for further functional characterisation. This bioinformatic analysis is typically reliant upon empirical threshold stringencies; however, several machine learning approaches have subsequently been developed, such as EffectorP (Sperschneider and Dodds, 2021) and Predector (Jones et al., 2021), to aid in effector identification (**Figure 1**).

Advances in RNA sequencing technologies like RNAseq have enabled effector predictions to be complemented with expression characteristics. Recent analysis of the expression profiles of known avirulence effector genes [*AvrM*, *AvrM14*, *AvrL2*, *AvrL567*, *AvrP123* (*AvrP*), *and AvrP4*] from the model flax-flax rust pathosystem (*Linum usitatissimum* - *Melampsora lini*) (Dodds et al., 2004; Catanzariti et al., 2006: Barrett et al., 2009; Wu et al., 2019) showed that *Avr* expression was greatest in haustoria and during infection and notably absent in germinated spores. Expression-based clustering grouped all cloned flax rust *Avrs* together, in addition to homologs of these genes present in *M. larici-populina* (Duplessis et al., 2011). Similar expression profiling also grouped together known *Pgt Avrs* (*AvrSr50*, *AvrSr27*, and *AvrSr35*) (Upadhyaya et al., 2021), suggesting expression patterns, particularly haustorial expression for rust pathogens, can be used as an additional criterion for candidate avirulence effector identification. However, additional functional assays are needed for avirulence effector confirmation.

Although the number of confirmed avirulence genes from *Pgt* is increasing, there still remains a paucity when compared with the availability of cloned *Sr* genes suitable for RTGS. Three *Avr* genes have previously been reported from *Pgt* (*AvrSr35*, *AvrSr27*, and *AvrSr50*) (Chen et al., 2017; Salcedo et al., 2017; Upadhyaya et al., 2021) and, very recently, a further two identified (Arndell et al., 2023). These latter two *Avrs*, *AvrSr13* and *AvrSr22*, were isolated using a high-throughput wheat protoplast assay coupled with deep sequencing. This new technology transforms an entire candidate effector library and R gene of interest into plant protoplasts. The recognized effector is detected by its transcriptional absence in a deep-sequenced protoplast RNA library due to cell death (Arndell et al., 2023).

In spite of their sequence diversity, effector proteins from filamentous pathogens can have remarkable similarity at a tertiary structural level, despite originating in pathogens from different taxonomic groups (reviewed recently by Outram et al., 2022). Recent developments in computational deep learning methods and large-scale, structure–structure comparison (Jumper et al., 2021; van Kempen et al., 2022) are heralding a new era in structural genomics. These approaches have proven informative in understanding the evolution of some key virulence effector proteins (Mark and Sylvain, 2023; Seong and Krasileva, 2023). Potentially structural genomics, in conjunction with other “omics” technologies, will facilitate novel avenues to identify effectors or reduce candidate numbers for downstream validation processes.

In addition to being valuable for confirming RTGS function, *Avrs* can provide highly accurate pathogen genotyping that will guide future informed deployment of RTGS in appropriate locations. Ideally, all *R* genes in a RTGS would function against all prevailing pathogen isolates globally, although the limited availability of such broad-spectrum *R* genes makes this goal difficult. More likely is that some RTGS genes will be ineffective against pathogens in some regions but not others, making it necessary to determine local pathogen virulence and avirulence profiles for optimal regional RTGS deployment. *Avr* identification promises to accelerate this process as simple PCR, and sequencing analyses can provide accurate pathogen genotypes. In addition, genotyping can provide some insight into the likelihood of future virulence evolution with homozygous *Avr* genotypes assumed less likely to evolve virulence than heterozygous individuals, as mutation of only a single allele is required for the latter genotype.

However, a platform for pathotyping-by-genotyping is still a long way off, and traditional pathology approaches will be still essential into the foreseeable future. Population studies on cloned effectors have highlighted the extensive genetic variation that can occur at these loci. In the case of *AvrSr50*, the analysis of a global collection of 38 *Pgt* isolates identified 14 alleles, with five alleles recognized by *Sr50* in transient expression assays and four not (Ortiz et al., 2022). Accurate pathotyping by DNA sequencing will require each new polymorphic effector allele to be tested for virulence/avirulence using transient assays if cognate R proteins are available and using pathology assays if not. Given the entirely artificial expression levels obtained in transient assays, pathology assays remain the *in planta* gold standard. However, ultimately, in the future, a comprehensive catalog of *Avr* effectors will enable pathogen virulence phenotypes to be inferred by nucleic acid sequencing of tissues from infected plants in the field (Hubbard et al., 2015).

## Current limitations of RTGS approaches

**Figure 2:**
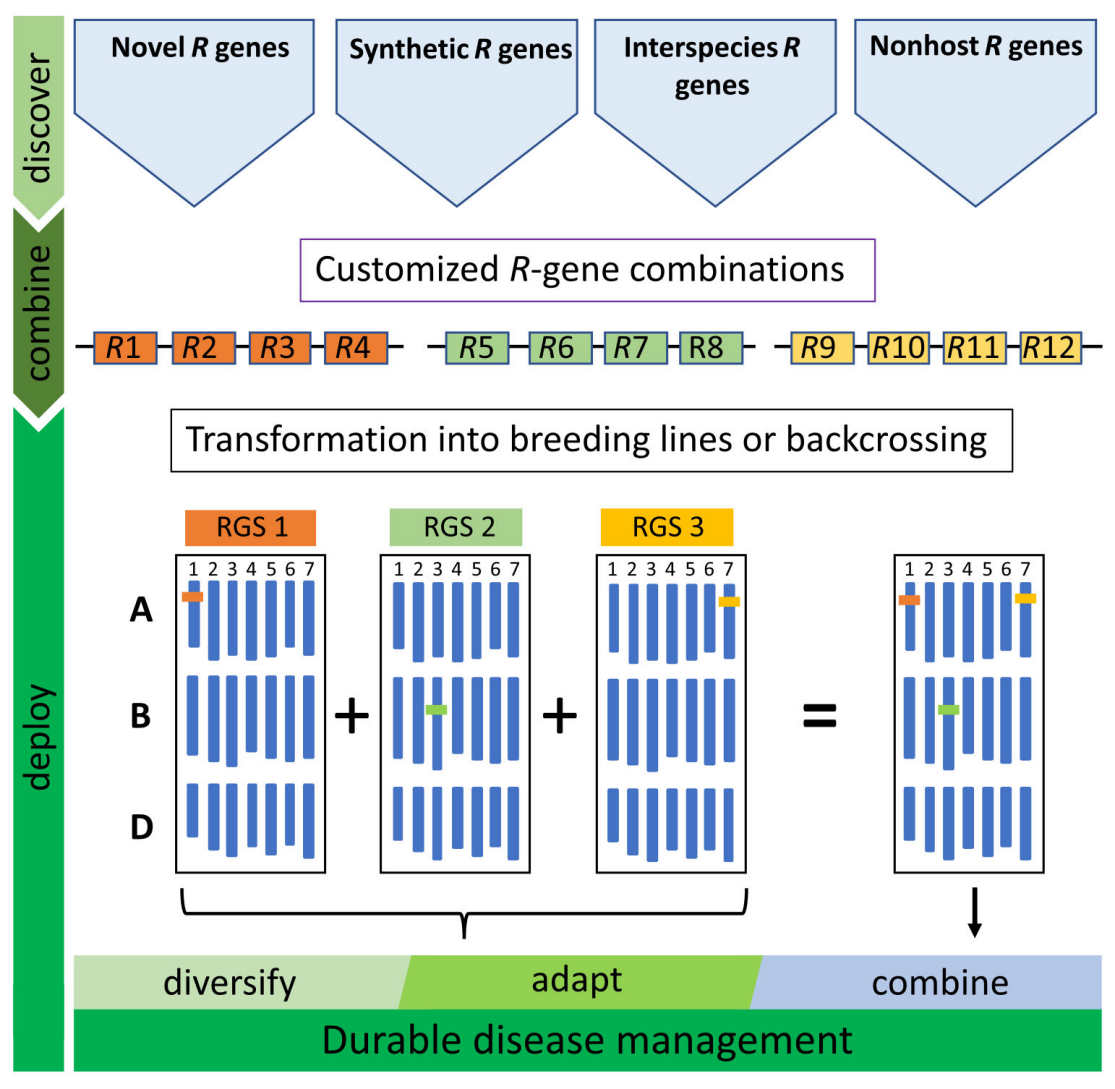
Deployment strategy of resistance gene stacks (RTGS). A range of resistance genes (*R*-genes) are needed to diversify RTGS for durable, broad-spectrum resistance. In addition to wheat *R* genes, genes can be sourced from land races, wild relatives, nonhost species or novel engineered genes (synthetic *R*-genes). Customized RTGS for regional or seasonal disease management can be rapidly assembled and integrated into breeding programs to adapt and react to newly evolving pathogen strains. The single locus inheritance of RTGS enables single or multiple cassettes to be used in breeding programs by backcrossing or alternatively direct transformation of elite wheat cultivars.

Although offering great potential for improving durable disease resistance, RTGSs do have limitations. For example, at the time of construction, all the *Sr* genes utilized in the first wheat RTGS (**Figure 1**, five-gene stack) were effective against east African isolate *Ug99* and many other *Pgt* isolates until the emergence of a new *Pgt* isolate (IT76a/18, race TTRTF) in Europe, which had overcome three of the four race-specific genes in the stack (i.e., *Sr35*, *Sr45*, and *Sr50*) (Luo et al., 2021). Deployment of this RTGS in Europe would, therefore, be equivalent to deploying a single race-specific resistance gene (*Sr22*) and a quantitative resistance gene (*Sr55*) against this race. However, until the emergence of the Digelu race in Ethiopia in 2019, and its subsequent migration across east Africa, this RTGS would have remained effective against stem rust in this region. Both examples highlight that regional rather than global deployment of RTGS will likely be a more successful approach and that overtime RTGS may need substitution. Pyramiding of several RTGS, however, by conventional breeding could reduce the necessity for regional deployment and further increase durability (**Figure 2**).

A potentially valuable use of RTGS would be to combine an allelic series from a single *R* locus to create a polygenic resistance that cannot be achieved by conventional breeding. For example, the wheat *Pm3* locus encodes a single powdery mildew *R* gene with 56 allelic variants, 17 of which confer resistance (Krattinger and Keller, 2016; Ayliffe et al., 2022). *Pm3* alleles have previously been functionally combined by crossing of single transgene lines (Koller et al., 2018). However, highly homologous sequences are unstable in the RTGS process, and two related resistance gene sequences, *Sr33* and *Sr50*, which are both members of the *Mla* gene family (Periyannan et al., 2013; Mago et al., 2015) and have 87% open reading frame (ORF) identity, could not be combined in *planta*. It should be noted, however, that two orthologous rice *NLR* genes, *Pi54* and *Pi54rh*, which have 93% nucleotide identity between ORFs, were successfully combined into a single construct and co-introduced into rice by biolistic transformation (Kumari et al., 2017). Another potential problem with a multi-allele gene stack is that, in some instances, alleles, or even orthologous genes (Hurni et al., 2014), can have cross-inhibitory effects which was observed for alleles of the wheat *Pm3* powdery mildew resistance gene with suppression occurring by a post-translational mechanism (Stirnweis et al., 2014). However, this problem could be avoided by judicious selection of compatible allele combinations (Stirnweis et al., 2014).

In addition, single-gene deployment of genes also used in a RTGS is likely to erode the efficacy of the stack over time. Gene deployment cannot be controlled for *R* genes used in conventional breeding making stacks including these genes potentially vulnerable. This problem could potentially be circumvented in wheat by using genes from wild relatives that are difficult to cross to wheat and that show poor recombination. Alternatively, effective genes could be sourced from more distantly related plant species that cannot be crossed to wheat (**Figure 2**), and these genes could potentially include components of non-host resistance (Bettgenhaeuser et al., 2014). These latter genes can only be introduced into wheat as transgenes and their deployment can be controlled by intellectual property rights.

Transferring RTGS into new genetic backgrounds also demands scrutiny to ensure that all genes remain functional in the particular genetic context. In wheat and other plant species, different genetic backgrounds can on occasion repress functional *R* genes (Bai and Knott, 1992; Kema et al., 1995; Nelson et al., 1997; Chen et al., 2013; Shandil et al., 2017; Gallois et al., 2018). In one case, in wheat resistance, suppression was caused by a gene encoding a subunit of the Mediator complex, which is a transcriptional coactivator (Hiebert et al., 2020). Genetic background, therefore, has the potential to suppress a member of an RTGS stack that would not be readily detected unless the function of all genes in the stack was reconfirmed prior to deployment.

Introducing a five-gene RTGS into wheat did not show any systematic influence on plant growth and yield parameter in glass house experiments nor cause obvious pleiotropic effects in disease field trials (Luo et al., 2021). However, a systematic assessment of multi-environment field trials is still needed to ensure that more subtle effects are not apparent. RTGS containing the same gene complement could potentially show differential pleotropic effects if located in different genomic locations. However, the extremely isogenic nature of RTGS lines and null sibs enables precise comparisons to be undertaken, far more so than for material containing multiple resistance genes distributed across the genome in recombined chromatin segments.

As described above, combining quantitative resistances into a RTGS is an attractive proposition given the polygenic nature of this resistance and assumed durability. However, the mechanistic basis of quantitative resistance is often not well understood, and not all partial resistance genes are additive. Broad-spectrum, multi-pathogen resistance genes such as *Sr55* and *Sr57* do not appear to function by effector recognition given the diverse range of pathogen species that they recognize (note that these two genes are not additive and, therefore, not candidates for combination; Lagudah and Holloway, unpublished). Given that the mechanistic basis of quantitative resistance is not well understood, it could be difficult to confirm that each quantitative gene in a RTGS is functional unless very clear, additive phenotypes were associated with each gene. Testing individual *QR* gene function using isolated pathogen molecules does not appear to be an option for this resistance class.

Finally, in some pathosystems, gene stacks may be an entirely inappropriate method for disease protection. Take for example blackleg disease of canola in Australia caused by the fungal pathogen *Leptosphaeria maculans*. This haploid pathogen has prolific asexual and sexual reproductive cycles and can rapidly overcome individual *R* genes (Rouxel et al., 2003; Sprague et al., 2006; van de Wouw et al., 2014). Strong sexual cycles allow pathogens to rapidly combine virulence alleles giving them the potential to overcome RTGS and resistance gene combinations produced by conventional breeding (McDonald and Linde, 2002). *L. maculans* virulence alleles exist for most available *R* genes, and combining these genes in a RTGS could promote the evolution of super-virulent isolates. This, in turn, would undermine other potential disease management strategies for this pathogen such as periodic single–*R* gene rotation (Marcroft et al., 2012; van de Wouw et al., 2014).

## Future gene stacks and deployment

Despite the advanced genomics technologies described above facilitating RTGS production, more resistance genes from many crop species are needed. Future RTGS will be dependent on an ever-increasing number of *R* genes becoming available or, alternatively, an ability to synthetically engineer new recognition specificities in *R* genes. For example, the wheat *Sr33* rust resistance gene was recently engineered to recognize an unrelated effector, *AvrSr50* (Tamborski et al., 2022). In addition, insertion of engineered nanobodies in NLR proteins containing integrated decoy domains has enabled new target molecule recognition to be achieved (Kourelis et al., 2023).

Future RTGS are likely to use targeted integration technologies such as gene editing to enable precise genomic insertions (Day et al., 2000; Ainley et al., 2013). This same technology may facilitate truly modular RTGS design, whereby defeated *R* genes may be removed and replaced with new alternatives. It may also facilitate removal of non-plant sequences from RTGS in the plant genome to produce plant only (cisgenic) insertions that have greater consumer acceptance and reduced regulatory burden. To date, cisgenic RTGS in potato that encode greater than two genes at a single locus have not been successfully produced. However, two gene stacks that are selectable marker-free and vector backbone–free have been developed (Jo et al., 2014; Haverkort et al., 2016).

Combining transgenes for genetic simplicity is not restricted to disease resistance and applicable to many other GM traits including insect resistance, herbicide resistance, metabolic engineering, and combinations thereof (Ye et al., 2000; Halpin, 2005; Petrie et al., 2014; Shehryar et al., 2019). Metabolic engineering raises new possibilities for disease protection given the long association between plant secondary metabolites and microbial resistance and the numerous efforts to exploit these molecules by enzyme overexpression and mutation (Grobkinsky et al., 2012; Jeandet et al., 2013). Gene stacking enables multi-enzyme pathway engineering with monogenic inheritance, as shown for plant production of new oil compounds (Wu et al., 2005; Petrie et al., 2014). This furthers the possibility of engineering in host plants non-endogenous secondary metabolite pathways not previously encountered by adapted pathogens.

Perhaps the greatest remaining obstacle for RTGS deployment in wheat is the consumer acceptance of this crop as a genetically modified organism (GMO) and the probable international regulatory differences that will exist between countries for this globally exported commodity. Whereas other genetically modified (GM) crops, such as cotton, corn, and soybeans, have been widely grown, globally wheat has been dubbed “the cereal abandoned by GM” (Wulff and Dhugga, 2018). However, recently commercial cultivation of the first GM wheat crop (HB4) has been approved in Argentina and Brazil. HB4 wheat has improved drought resistance, showing yield improvements of ~20% compared with their non-GM counterparts under water-limited conditions. This GM wheat deployment heralds a marked shift in attitude, and the future may see increasing deregulation of GM wheat varieties. Of further interest is the (7 CFR part 340) regulatory exemption granted by the United States Department of Agriculture - Animal and Plant Health Inspection Service (USDA-APHIS) for a potato line produced by US company J. R. Simplot that carries three *P. infestans* resistance genes: (*Rpi*) *Rpi-vnt1*, *Rpi-amr3*, *and Rpi-blb2*, in addition to viral resistance and several quality traits all encoded on a 30-kb T-DNA sequence (https://www.aphis.usda.gov/brs/pdf/rsr/21-270-01rsr-review-response.pdf).

Annually, global crop losses of 10%–30% are caused by pests and diseases (Savary et al., 2019), despite the application of four million tonnes of pesticides and fungicides (Steinburg and Gurr, 2020; Pathak et al., 2022). With a requirement to increase food production by at least 60% by 2050 (van Esse et al., 2020), it is clear that innovative approaches will be required to meet this demand. RTGS represent one such technology that could be highly beneficial in achieving this goal, in addition to helping to reduce the chemical reliance of broad-scale agriculture.

## Author contributions

All authors contributed to the article and approved the submitted version.
